# Antibodies to CD20 and MHC class II antigen bound to B-lymphoma cells accumulate in shed cytoplasmic fragments

**DOI:** 10.1038/sj.bjc.6602131

**Published:** 2004-09-28

**Authors:** R B Michel, M J Mattes

**Affiliations:** 1 Center for Molecular Medicine and Immunology, 520 Belleville Avenue, Belleville, NJ 07109, USA

**Keywords:** antibody therapy, antibody processing, shedding, B-cell lymphoma, CD20 antigen, histocompatibility antigens class II

## Abstract

Antibodies (Abs) to CD20 and MHC class II antigen were found to exhibit a novel processing pathway after binding to the surface of RL B-lymphoma cells. The Abs were ‘excreted’ as a part of large cytoplasmic fragments. These fragments formed at cell–cell junctions, but gentle dispersal of the cells, to form a single-cell suspension of high viability, caused the release of most of the fragments. This process also occurred in Raji cells and in three other B-lymphoma cell lines (of seven tested). Six B-lymphoblastoid cell lines tested did not form these objects. Once they were recognised, the fragments could be identified in cell preparations by phase contrast microscopy or after staining with Wright's stain. They were induced by the binding of certain Abs, but not by most Abs bound to the cell surface. The mode of formation, detailed morphology and function of these cytoplasmic fragments remain to be determined. They are similar in many respects to the lymphoglandular bodies that have been described by pathologists for many years, which are characteristic of B-cell lymphoma, but which have not previously been described in cell lines. This type of Ab processing, if it occurs in patients, will have an impact on the therapeutic use of these Abs.

CD20 is a B-cell-specific marker that has been used extensively as a target for antibody (Ab) therapy in patients with B-cell lymphoma. Unconjugated Abs have become established as standard therapy (McLaughlin *et al*, 1998), and radiolabelled Abs, conjugated with ^90^Y, are significantly more effective than the unconjugated Abs (Witzig *et al*, 2000). The processing of the Ab after binding to the cell surface is an important factor that will affect its therapeutic activity. We recently investigated the fate of anti-CD20 Abs after binding to the surface of B-lymphoma cell lines (Michel and Mattes, 2002). Using anti-CD20 (1F5) conjugated to the fluorophore Alexa 488, the Ab was found to gradually accumulate in a noncatabolic intracellular site. This site had the appearance of a large juxtanuclear (JN) spot in Raji and Daudi cells, while in Ramos cells the fluorescence was more dispersed, and consisted of smaller cytoplasmic vesicles. In all three of these cell lines, the location of the Ab was virtually identical to the steady-state localisation of transferrin, as seen with a simultaneous rhodamine label. These data indicate that anti-CD20 localises to the endocytic recycling compartment (ERC), although its rate of transfer to this compartment is much slower than that of transferrin. This result was unexpected, in that anti-CD20 is widely considered to be a noninternalising Ab; this apparent discrepancy can be explained by the fact that intracellular accumulation of the Ab is slow, and only prominent after many hours of incubation (generally overnight).

The RL cell line differed from the other three cell lines tested, in that prominent cytoplasmic accumulation of the Ab was not evident, although initial cell surface binding was as strong as with the other cell lines. In addition, long-term saturation experiments with radiolabelled Ab revealed an apparent paradox: total uptake of Ab per cell was much higher at 24 h than at 1 h, by a mean factor of 2.5 (unpublished data), similar to the increase described previously with Raji cells (Ong *et al*, 2001; Michel and Mattes, 2002). Since such Ab uptake in RL cells was not detected by immunofluorescence (by FACS or microscopic analysis), the implication of these results is that the Ab was taken up by RL cells at some site that was not seen by immunofluorescence. In the current study, we examined Ab uptake in RL cells more thoroughly, using, initially, a more sensitive assay, namely immunoperoxidase staining. By this method, and subsequently by immunofluorescence, it was found that anti-CD20 Abs, and certain but not all other Abs, accumulated in large shed cytoplasmic fragments after binding to the cell surface. Production of these cytoplasmic fragments was induced by Ab binding, and occurred in most, but not all, B-lymphoma cell lines, to varying degrees; it did not occur in B-lymphoblastoid cell lines.

The function of the CD20 antigen is not fully known, but it appears to play a role in lymphocyte activation, and evidence suggests that it may function as a Ca^2+^ channel (Bubien *et al*, 1993). It is a tetraspan membrane protein, with only a small loop present on the exterior of the cell (Zhou and Tedder, 1995). All Abs recognise a single epitope, in competitive binding experiments, although different Abs differ in their physiological effect (Zhou and Tedder, 1995). Antibody binding induces the rapid redistribution of nearly all of the CD20 to a low-density, detergent-insoluble plasma membrane compartment (Polyak *et al*, 1998; Semac *et al*, 2003).

## MATERIALS AND METHODS

### Cell lines, Abs and Ab conjugates

The B-lymphoma cell lines Raji, Ramos, and Daudi and the lymphoblastoid cell lines RPMI-1788, RPMI-7666, IMP-9, SKW6.4, GK-5 and EH IV were obtained from The American Type Culture Collection (ATCC, Rockville, MD, USA). The RL cell line, a diffuse, large-cell B-lymphoma cell line, was described previously (Hansen *et al*, 1996), and is now available from ATCC. SU-DHL-4 and SU-DHL-6 cell lines (Epstein *et al*, 1978) were provided by Dr AL Epstein (University of Southern California Medical Center, Los Angeles, CA, USA). The BJAB cell line (Schmid *et al*, 1993) was obtained from Dr ES Vitetta (University of Texas Southwestern Medical Center, Dallas, TX, USA). Cells were cultured as described (Hansen *et al*, 1996; Ong *et al*, 1999). They were tested routinely for mycoplasma by the Mycotect assay (Life Technologies, Grand Island, NY, USA), and were negative. All of the Abs used were described previously (Hansen *et al*, 1996; Ong *et al*, 1999). Anti-CD147 (MA103) is produced by a hybridoma generated by us. Anti-CD20 Abs (1F5 and 2B8) and anti-HLA-DR Abs (L243 and IVA12) were produced by hybridomas obtained from ATCC. In both cases, the Ab subclasses are IgG2a and IgG1, respectively. Antibody 2B8, as obtained from ATCC, was found to consist of primarily non-Ab-producing cells; therefore, Ab-producing cells were cloned by limiting dilution before expansion. Rituximab is a human/mouse chimeric Ab to CD20, derived from 2B8, which is a product of IDEC Pharmaceuticals, and was purchased from Florida Infusions (Palm Harbor, FL, USA). Anti-CD22 (LL2) and anti-CD74 (LL1) Abs were provided by the Antibody Production laboratory of Immunomedics, Inc. (Morris Plains, NJ, USA). Anti-CD19 (HD37) was provided by Dr Ellen Vitetta. Control Abs of the same subclasses were also tested, namely the IgG1 Ab MOPC-21 and the IgG2a Abs TA99 and MX352a. Rabbit anti-human IgM was purchased from DAKO Corp. (#A425, IgG fraction, Carpinteria, CA, USA). Both anti-CD20 Abs, anti-CD147, and a control Ab, MX352a, were conjugated with Alexa 488, from Molecular Probes (Eugene, OR, USA) as described previously (Michel and Mattes, 2002). Preparation of the Fab fragment of Ab 1F5, and its conjugation with Alexa 488, was described previously (Michel and Mattes, 2002). Antibodies were sterilised by passage through a 0.2 *μ*m pore size filter. Peroxidase staining utilised reagents from Vector Laboratories (Burlingame, CA, USA), namely biotinylated horse anti-mouse IgG, and a complex of streptavidin and biotinylated peroxidase. For use with the rabbit anti-human IgM antisera, we used a biotinylated anti-rabbit IgG from Vector Labs.

### Immunoperoxidase staining

Cells (2.5 × 10^5^) were incubated overnight in a well of a 24-well plate in 0.5 ml medium containing Abs at a concentration of 10 *μ*g ml^−1^. Cells were then transferred to a tube, pelleted, suspended in 2.5 ml of DPBS, 0.5% BSA, 10 mM NaN_3_, and cytocentrifuge slides were prepared, with a StatSpin Cytofuge 2 (Norwood, MA, USA), using 0.2 ml per slide. After air drying, slides were fixed with 4.0% formaldehyde in phosphate-buffered saline (PBS) for 20 min at room temperature. The formaldehyde was prepared from depolymerised paraformaldehyde as described (Farr and Nakane, 1981). After washing with DPBS, slides were flooded and incubated for 10 min with DPBS-BSA-N_3_ with or without 0.15% saponin. All buffers used for washes and incubations in subsequent steps were also either with or without saponin, as appropriate. After discarding the buffer, the spot of cells was covered with 50 *μ*l biotinylated horse anti-mouse IgG (Vector Labs), diluted 1 : 200, in the same buffer. For use with the rabbit anti-mouse IgM, biotinylated anti-rabbit IgG was used. After 1 h incubation at 37°C in a humid chamber, slides were washed once rapidly and 2 × 10 min with the same buffer. The cell spot was then covered with 50 *μ*l ABC reagent (Vector Labs) and incubated 1 h at 37°C. After washing as above, the cell spot was covered with 0.1 ml DAB solution, prepared as follows. A 10 mg tablet of DAB (Sigma Chemicals, St Louis, MO, USA) was dissolved in 20 ml 0.05 M Tris–HCl, pH 7.2, and 6.6 *μ*l of 30% H_2_O_2_ was added. Saponin was not included in this or subsequent steps. After 15 min at room temperature, slides were washed three times with PBS, once with water, and air-dried. They were counterstained for 5 min with Gill's haematoxylin (#GHS-1-32, Sigma Chemicals), washed with water, air dried, and a coverslip was mounted with Cytoseal (VWR, West Chester, PA, USA).

### Immunofluorescence

Cells were generally incubated overnight with the directly conjugated Ab at 10 *μ*g ml^−1^. The Fab fragment was used at 50 *μ*g ml^−1^. After two washes, they were examined in an Olympus fluorescent microscope with a 100 W mercury bulb. In some experiments, smears were prepared, after suspending the cells in a small volume of foetal bovine serum. The smears were fixed in methanol for 10 min, and examined with a loose coverslip. After photographing cytoplasmic fragments, and recording the *X* and *Y* coordinates of the slide holder, the slide was stained with Wright's stain, and the same cells were then re-examined. In some experiments, cells were examined without washing away unbound Ab, by transferring the cell suspension gently (so as not to disperse cell clusters) directly to slides for observation. Representative samples were examined with a Zeiss LSM 410 confocal microscope, equipped with an argon–krypton laser.

### Other methods

Wright's staining was by standard methods, using stain from Harleco (#740, purchased from VWR, West Chester, PA, USA). Photographs were taken with an Olympus Microfire digital camera.

## RESULTS

### CD20-containing cytoplasmic fragments from RL cells

**Figure 1:**
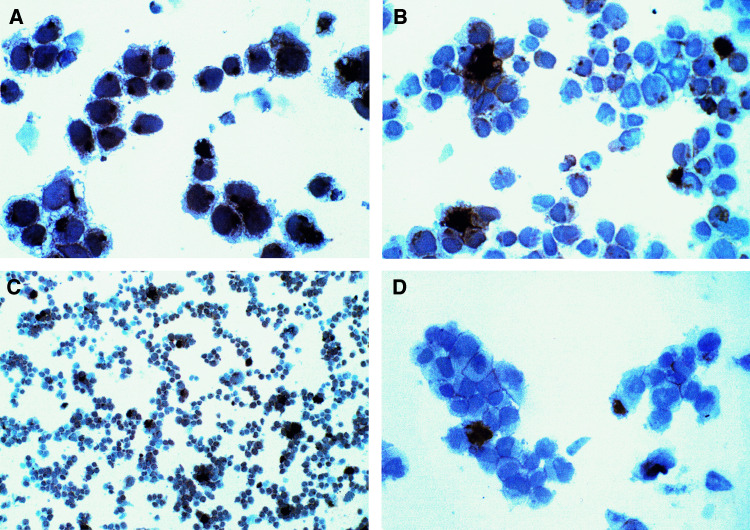
Immunoperoxidase staining of B-lymphoma cells after overnight incubation with anti-CD20 (1F5). Cells were deposited on cytocentrifuge slides, fixed with formaldehyde, permeabilised with saponin, and stained with a biotinylated horse anti-mouse IgG, followed by a complex of streptavidin and peroxidase. Observation was with an × 40 objective, except as noted. (**A**) Raji cells, showing prominent staining of JN spots. (**B**) RL cells, showing dark staining of apparently extracellular objects, referred to as CFs. (**C**) A lower-power photograph of RL cells (× 10), to show the general staining pattern. (**D**) RL cells stained in the absence of saponin, to show antigen that is accessible without permeabilisation. Control Abs of the same subclass produced no brown staining.

The immunoperoxidase staining method was intended to provide greater sensitivity than the immunofluorescence assay, and to thereby reveal the location of anti-CD20 Abs taken up by RL lymphoma cells. Raji cells were used in preliminary experiments, to develop the assay, since it was previously established, by fluorescence, that anti-CD20 localised to a JN site in this cell line. This same pattern of localisation in Raji cells was demonstrated by the peroxidase method (Figure 1A

). In RL cells, in contrast, cytoplasmic peroxidase staining was much less prominent, although small cytoplasmic vesicles were stained (Figure 1B). However, large, apparently extracellular objects were very darkly stained in RL cell preparations (Figure 1B, C). Some of these were as large as cells, or larger, but they were heterogeneous in size, and some were much smaller. The total number of these objects was approximately 5% of the number of cells. Due to the very dark peroxidase staining, the nature of the objects that were stained could not be distinguished. Therefore, additional experiments were performed in which peroxidase staining was made lighter, by simply decreasing the time of the peroxidase reaction. From these studies, it was found that the stained objects appeared to be large cytoplasmic fragments, not containing a nucleus. Most of these objects appeared to be bound to the outer surface of cells, but some were unattached. We note that the cytocentrifuge method used tends to cause extensive clustering of cells that are not really clustered, as the cells are deposited on the slide. Therefore, the fact that the stained objects were often within cell clusters does not indicate that such aggregates were present initially. We tentatively refer to these objects as cytoplasmic fragments (CFs).

Staining experiments were also carried out in the absence of saponin, in which case the Ab should be unable to cross membranes. The CFs were also stained in this experiment, but much less darkly than in the presence of saponin (Figure 1D). Staining was variegated, with some areas of the CFs much more darkly stained than others. We conclude that some of the Ab in the CFs is not enclosed in a membrane. But since the staining was much weaker than in the presence of saponin, the majority of the bound 1F5 is apparently within a membrane-enclosed compartment. In the experiment without saponin, there was no staining of cytoplasmic vesicles, as expected. There was sharp cell membrane staining in the absence of saponin (Figure 1D), but only approximately 10% of the cells had this membrane staining, which we cannot explain, but presumably is due to a problem of accessibility and/or fixation. The membrane staining was sharper in the absence of saponin than in its presence, probably because saponin caused some disruption of the cell membrane.

**Figure 2:**
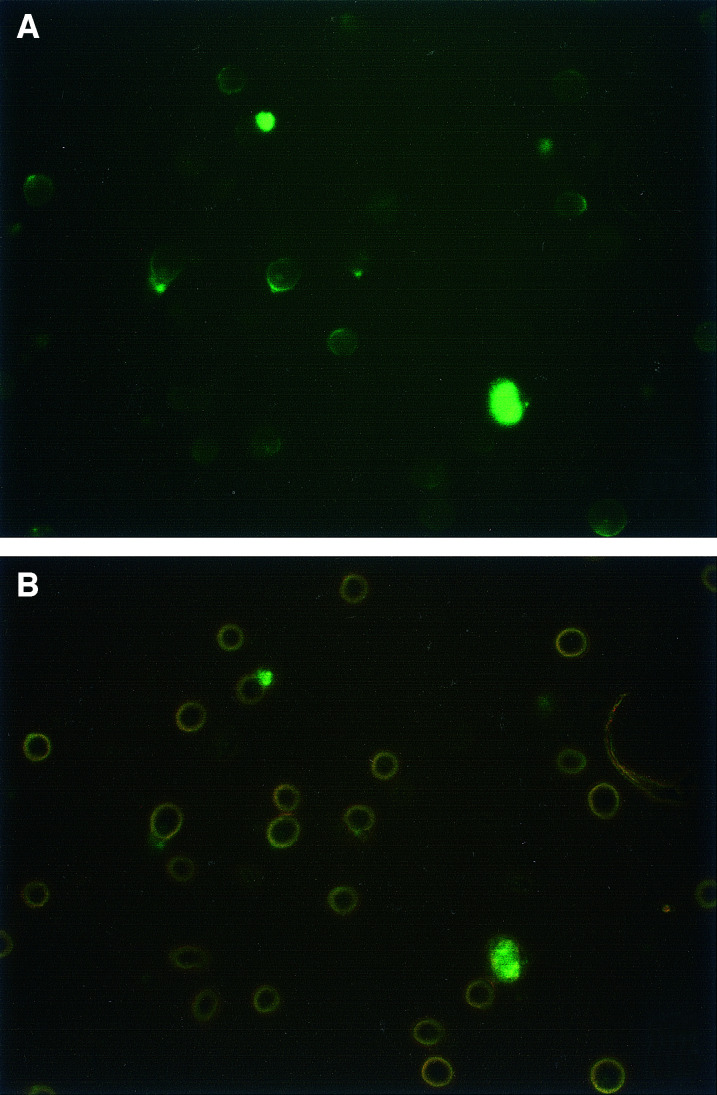
Fluorescence of RL cells after overnight incubation with Alexa 488-1F5. (**A**) Fluorescence, showing one large unattached CF and a smaller cell-bound CF, as well as weaker staining of the membrane and cytoplasmic vesicles of most of the cells. The CFs are overexposed, in order to allow membrane staining to be seen. (**B**) Dim visible light (using phase contrast) together with fluorescence, showing the same cells. Observation was with an × 40 objective.

**Figure 3:**
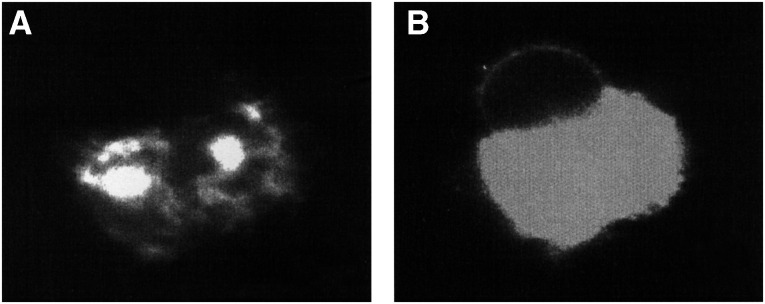
Appearance of a CF on a confocal microscope. RL cells were incubated overnight with Alexa 488-1F5; a large CF attached to a cell is shown. (**A**) The pinhole size was 20, giving a slice thickness (FWHM) of 1.5 *μ*m. (**B**) The pinhole size was 50, giving a slice thickness of 3.6 *μ*m, and the laser power was increased to allow cell membrane staining to be seen.

To further characterise these darkly stained objects, similar experiments were performed by immunofluorescence, using a directly labelled anti-CD20 Ab. As shown in Figure 2

, similar very brightly stained extracellular objects were seen by fluorescence. Again, some of these were attached to cells, and others were unattached. Since these cells were examined in suspension, the artefactual clustering that occurs in cytocentrifuge preparations was not a factor. In addition to the very bright CFs, the cell membrane and small cytoplasmic vesicles were more weakly stained, with most of the membrane staining having a cap-like appearance, as shown (Figure 2A). Early time points from 1 to 5 h were examined, in an attempt to determine the origin of the CFs. At these early time points, staining was heterogeneous, with some cells stained in a ring pattern, but most being partially capped. Small caps, as occur with strongly capping Abs such as anti-IgG (Schreiner and Unanue, 1976), were not observed at any time point. Fluorescent CFs were first visible at 4–5 h, but increased in frequency and size with time, and became much more prominent after overnight incubation with the Ab. By visible light, using phase contrast observation, the CFs were clearly different from cells, so could be recognised by visible light only. They were irregular in shape, nonrefractile (unlike viable cells) and appeared to contain smaller vesicles. Fluorescent staining of the CFs was variegated, as shown in Figure 2B. Cytoplasmic fragments were not seen when cells were incubated with a control, nonreactive Ab of the same subclass, conjugated in the same way. Moreover, cell preparations incubated with control Abs or without Abs did not contain CFs that could be recognised by visible light, indicating that the CFs were induced by Ab binding. As an additional specificity control, cells were incubated with Alexa 488-MX352a (a nonreactive Ab) together with unconjugated 1F5, at 10 *μ*g ml^−1^. This experiment was intended to eliminate the possibility that CFs are induced by 1F5, and then stain nonspecifically with any Ab conjugate. This control also was negative. To obtain better resolution of the CFs, representative samples were examined on a confocal microscope. This displayed the variegated staining pattern more clearly, showing unstained areas as well as brightly stained areas within the same CF (Figure 3A

). The CFs contained vesicle-like objects, having smooth, sharp boundaries, some of which were stained, and others unstained. Those that were unstained often appeared to have a fluorescent membrane, or to be surrounded by diffuse fluorescent material within the CF.

Free and cell-bound CFs were counted: in one typical case, after overnight incubation with 1F5, 65 of 102 CFs (64%) were unattached to cells. Cells were sometimes counterstained with propidium iodide, to identify dead cells. Cells having attached CFs were virtually all viable cells. Also, the CFs themselves were unstained by propidium iodide, indicating that they contain no nucleus or nuclear fragments. A second Ab to CD20, rituximab, was also conjugated to Alexa 488, and tested for comparison: the staining pattern on RL cells after overnight incubation was generally very similar to that of 1F5, although staining appeared to be slightly brighter with rituximab. Immunoperoxidase experiments were also performed with a second Ab to CD20, 2B8 (the mouse IgG1 Ab that was the origin of rituximab): again, staining was essentially the same as with 1F5. Since the CFs are heterogeneous in size, it seemed possible that small, cell-free CFs might be missed in our experiments, if they are too small to pellet under our normal washing conditions. To investigate this possibility, the supernatant obtained after the normal wash of Ab-treated cells was subjected to a spin for 30 min at 10 000 r.p.m. No visible pellet was obtained, and no CFs were detected in the pellet by microscopic observation. We conclude that essentially all CFs are large enough to pellet with the cells.

**Figure 4:**
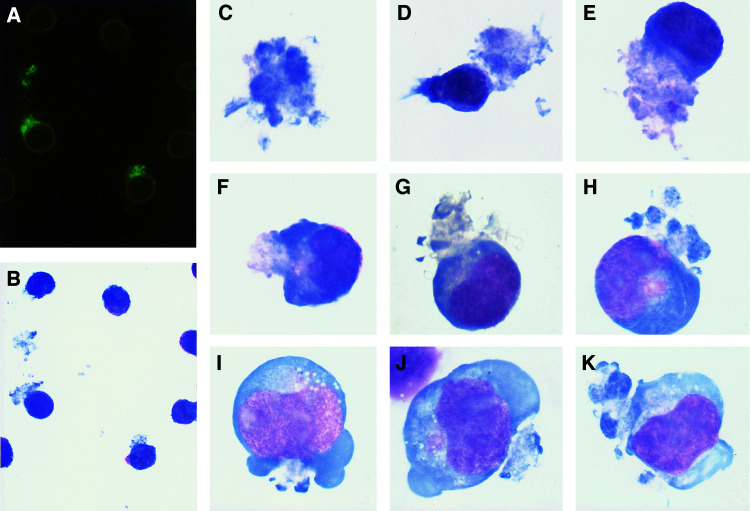
Appearance of CFs from RL cells (**A–F**) or Raji cells (**G–K**) after staining with Wright's stain. All CFs shown were first identified by fluorescence, after overnight incubation with Alexa 488-1F5, on smears fixed with methanol. The location (*X* and *Y* coordinates) of fields containing typical fluorescent CFs was recorded, photographs were taken, and the same objects were examined after Wright's staining. (**A**) An example of fluorescent CFs, showing one unattached and two cell-bound CFs. Observation was with dim visible light as well as fluorescence, in order to allow the cells to be seen. Essentially all of the cells also had weaker staining of the cell membrane and cytoplasmic vesicles, as shown in Figure 2A, but this staining is not visible at this exposure setting. (**B**) The same field stained with Wright's stain. (**C**) An example of a cell-free CF. (**D–K**) Examples of CFs that appear to be in the process of detachment from cells. In all cases, the fluorescence was over the part of the cell that was identified morphologically as a CE, as exemplified in Figure 3A. Observation was with an × 40 objective for (**A**, **B**), and an × 100 objective for the other photographs.

To better characterise the morphology of these objects, they were examined using Wright's stain, after being first identified by immunofluorescence. Cells were stained overnight with fluorescent anti-CD20, then used to prepare smears, which were fixed with methanol. Fluorescence remained bright in such preparations, and CFs could be readily identified. They were photographed, and the *X* and *Y* coordinates of the slide holder were recorded. The slides were then stained with Wright's stain, and the same CFs were re-examined. Figure 4

shows the appearance of representative CFs. As a demonstration of the method used, Figure 4A shows an example of the fluorescent staining of two cell-bound CFs and one free CF, and Figure 4B shows the same cells after staining with Wright's stain. Note the variegated fluorescent staining of the CFs. All of the other cells or CFs shown in Figure 4C–K were, similarly, identified initially by their bright fluorescence. Figure 4C shows a typical free CF, which is the most common type of CF in these preparations. In general, the CFs stained with Wright's stain looked like cytoplasmic fragments, but with variegated blue staining: some areas were relatively dark blue, and others were very pale, often with a slight pink tinge. They typically appeared to contain some fibrous elements. A clear relationship between structures stained by fluorescence and Wright's stain was not apparent. Another common type of CF seen was bound to a cell, but apparently separate from that cell. Cytoplasmic fragments that appeared to be ‘budding’ from the cell, although relatively rare, are of particular interest, since these provide some indication of the process by which CFs are generated. Figures 4D–K, were chosen to shown examples of the polymorphic ‘budding’ cells; Figures 4D–F show RL cells, and Figures 4G–K show Raji cells, which produced generally similar CFs, as described below. In all cases, bright staining was localised to the CF and not to the normal part of the cell. As shown, the relationship between the cell and the ‘budding’ CF is complex. The nature of this budding process remains to be characterised, and electron microscopy is required to fully analyse the process. In some Raji cells (Figure 4H, J and K), the CFs seemed to be ‘budding’ from more than one location on the cell surface. Many of the CFs were comparable in size to the cells to which they were attached, which seems difficult to explain, but it should be considered that the CFs may be produced not by a single cell, but by a cluster of cells, a possibility which is strongly supported by data presented below. After examining CFs in these experiments, we were able to readily identify CFs by Wright's staining alone, in the absence of fluorescent staining.

### Are other Abs delivered to CFs?

Using the immunoperoxidase method, similar experiments were performed with six other Abs, namely mouse mAbs to CD19, CD22, CD74, CD147, HLA-DR and rabbit anti-human IgM. Only one other Ab stained CFs darkly, anti-HLA-DR (Ab L243). Such staining was even darker than with anti-CD20 (data not shown). L243 also stained JN spots and the cell surface, but this staining was much weaker than the staining of CFs. Staining with an IgG1 Ab to HLA-DR, IVA12, produced results very similar to those obtained with L243 (which is an IgG2a). Other Abs did not stain CFs at all (CD19, CD22, CD74, anti-human IgM), or weakly stained a small fraction of CFs (CD147). All of these Abs stained RL cells, and some stained the cell surface as brightly or brighter than 1F5.

To determine whether crosslinking was required to induce CF formation, experiments were performed with the Fab fragment of 1F5, conjugated to Alexa 488. The Fab fragment also was delivered to CFs, which were very similar to those induced by the intact Ab, although the intensity of fluorescence in the CFs was considerably lower, and the CFs appeared to be somewhat smaller. We conclude that crosslinking by Abs is not required to induce CF production.

### Are CFs induced by Ab binding?

The immunofluorescence experiments described above indicated that CFs are induced by Ab binding, and are not present in control cells. To confirm this conclusion, smears stained with Wright's stain were examined, since CFs have a characteristic morphology that can be identified by Wright's staining alone, as noted above. Since CFs are rare, it was necessary to scan a large number of cells, at least 500. In three experiments, the number of morphologically distinct CFs in RL cell preparations, after overnight incubation with 1F5, was 0.8–1.9% of the cell number. This included both CFs that were cell bound, and CFs that were unattached to cells. There were no CFs present in control cell preparations. Cytoplasmic fragments identified solely by Wright's staining were also induced by anti-HLA-DR, but not by Abs to CD22, CD74, or human IgM, all of which react with the surface of RL cells. We conclude that the formation of CFs is induced by the binding of particular Abs.

### Production of CFs by other B-lymphoma and B-lymphoblastoid cell lines

Six other B-cell lymphomas and six B-lymphoblastoid cell lines were examined for the presence of CFs by both fluorescence and peroxidase methods. A list of the cell lines tested is given in Materials and Methods. All were tested with 1F5, and other Abs were also tested on some of the cell lines. The results of both types of assays were consistent, except as noted. After overnight incubation, 1F5 localised to cytoplasmic vesicles in all of the cell lines tested. Cytoplasmic fragments induced by 1F5 were clearly seen in four of the B lymphomas, but were not detected in any of the B-lymphoblastoid cell lines. Raji cells had the highest expression of CFs, of the new cell lines, and the appearance of CFs ‘budding’ from Raji cells, stained with Wright's stain, is shown in Figure 4. Figure 1A also shows three CFs from Raji cells: one in the centre, one at the upper right, near the edge, and the third at the lower right, close to the ‘A’. BJAB, SU-DHL-4 and SU-DHL-6 cells had low levels of CFs that were not as prominent. The number of CFs in Raji cell preparations was comparable to that with RL cells, but the size of the CFs was smaller. The lesser prominence of Raji CFs compared to those of RL cells was mainly due to the dark, homogeneous staining of JN spots in Raji cells (Figure 1A), which competes for attention. Raji cells were stained with all of the six Abs that had been tested on RL cells, and a control, nonreactive Ab, by the peroxidase method; and were also stained with and without saponin. Raji cells were also tested by immunofluorescence, using all of the Alexa 488 conjugates that had been tested on RL cells, including the conjugate of the Fab fragment of 1F5. All results were essentially the same as those obtained with RL cells, except that the JN spot was stained by Alexa 488-anti-CD20 in Raji cells, as described previously (Michel and Mattes, 2002) (in addition to the staining of CFs). The only other significant difference was that anti-CD147 stained CFs from Raji cells, about as darkly as 1F5, as seen with both peroxidase and immunofluorescent staining. Since Alexa 488-anti-CD147 did not stain a JN spot in these cells, the transport of Abs to CFs apparently does not require accumulation of the Ab in the ERC, suggesting that Ab internalisation may not be required. Also, while Alexa Fab of 1F5 still induced and stained CFs in Raji cells, quite similar to those induced by the intact Ab in abundance and staining intensity, and indistinguishable by morphology, the Fab fragment produced very little staining of the JN spot, as described previously (Michel and Mattes, 2002). These results suggest that formation of Ab-containing CFs by Raji cells is independent of the accumulation of the Ab in a JN spot. The lymphomas that did not produce CFs were Daudi and Ramos. Daudi was stained with the same panel of six Abs that was tested on RL and Raji cells: CFs were not detected with any of the Abs, all of which stained the cell surface and/or cytoplasmic vesicles. We note that the two B-lymphoma lines with no CFs are also the two lines that normally have the lowest level of clustering.

Although the lymphoblastoid lines did not display CFs, all were stained by anti-CD20. The staining was of both the cell surface and cytoplasmic vesicles, and was seen by both fluorescence and peroxidase methods. Cytoplasmic staining was similar to the staining pattern seen in Raji in some of these lines (RPMI-1788 and EH IV), with a JN spot; and similar to Ramos in other lines (IMP-9, SKW6.4, GP5, RPMI-7666), with a larger number of small, more dispersed cytoplasmic spots.

### Appearance of CFs in undisrupted cell clusters

**Figure 5:**
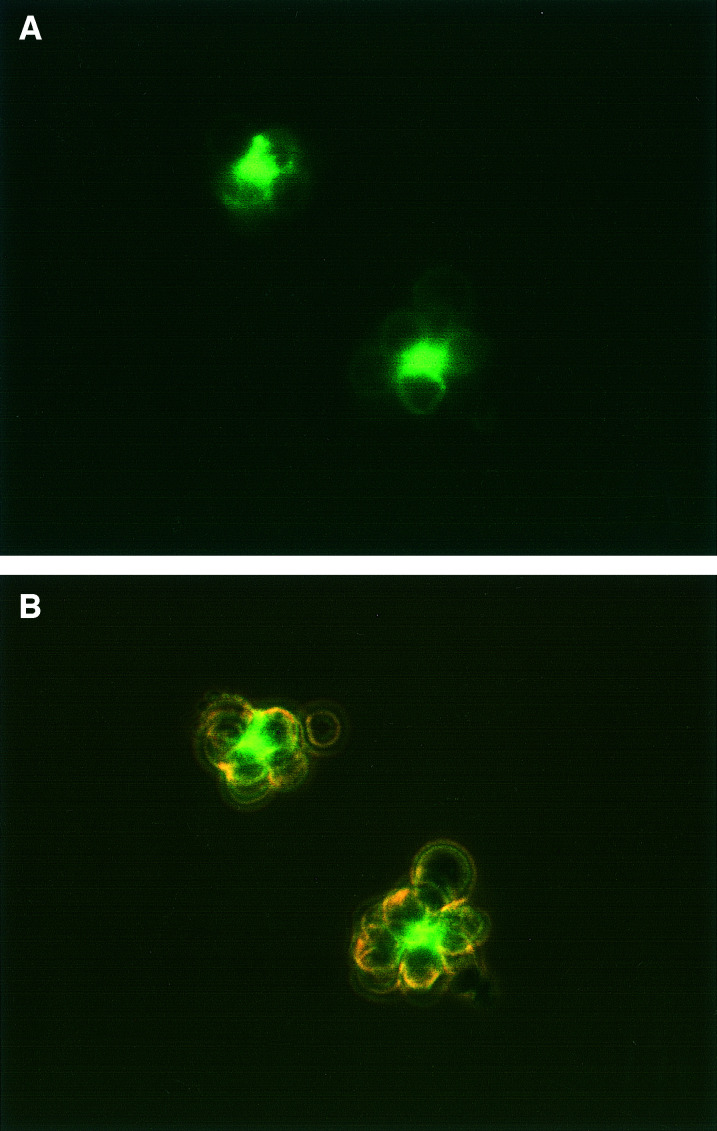
Fluorescence of RL cells after overnight incubation with Alexa 488-1F5. Cells were examined without washing away unbound Ab, and were handled as gently as possible, to leave cell clusters intact. (**A**) Fluorescence. (**B**) Dim visible light (using phase contrast) together with fluorescence, showing the same cells. Fluorescence is concentrated in the centre of a cluster of cells. Control Ab conjugates of the same subclass produced no significant staining. Observation was with an × 40 objective.

Most B-lymphoma cell lines, including RL, Raji, and BJAB, grow normally in large clusters, as do most of the B-lymphoblastoid cell lines. With RL and Raji cells (but not with BJAB cells), these clusters are readily dispersed by gentle pipetting and handling, so when the cells are washed, after Ab incubations, a single-cell suspension is obtained. Cytoplasmic fragments in undisrupted clusters could be examined by fluorescence, because the specific staining is so bright that it can be clearly seen without washing away the unbound fluorescent Ab. In these experiments, after overnight incubation with the Ab, the cells were transferred, as gently as possible, directly to a slide for observation. The appearance of RL cells in this type of experiment is shown in Figure 5

. The staining was almost entirely at the centre of the clusters, and no cell-free CFs were observed. We conclude that CFs are normally present only at the centre of cell clusters, and that they are separated from cells only when the cells are dispersed, by pipetting. With Raji cells, there was also staining at the centre of cell clusters, but it was much less prominent than with RL cells, mainly due to the bright staining of JN spots. Again, no cell-free CFs were detected unless cell clusters were dispersed. These results suggest that CFs form within cell clusters, and that the appearance seen previously in RL and Raji cell preparations is a consequence of mechanical disruption of the clusters. This interpretation can explain the variation in the size of the CFs, and the fact that the number of CFs is much less that the number of cells. That is, since most or all of the clustered cells appeared to be adjacent to a CF, at the centre of the cluster, it seems likely that all contributed to the formation of the CF. However, when the cells were dispersed by pipetting, the CFs usually detached from the cells, resulting in either a free CF or a CF remaining attached to only one cell.

## DISCUSSION

We have described a novel pathway taken by Abs binding to the surface of B-cell lymphomas, transport to large extracellular cytoplasmic fragments. They are formed only after the binding of certain Abs, and these Ab accumulate in these structures in large amounts. Thus, after overnight incubation of RL cells with anti-CD20, the great majority of the bound Ab has been transported to these CFs. If this type of Ab transport occurs in patients with B-cell lymphoma, it would have an impact on the use of radiolabelled anti-CD20 for cancer therapy, since the shedding of bound Abs would tend to reduce the accumulation of radioactivity in the cells. Inasmuch as cell debris may bind Ab nonspecifically, it is important to emphasise the specificity controls that were performed: the CFs were not stained by a nonreactive, subclass-matched fluorescent Ab, even in the presence of an unconjugated anti-CD20 Ab, which induces the production of CFs. In addition, viability remained high (>95%) throughout these experiments, so formation of CFs was not associated with cell death or deterioration. Since CFs were observed in cultured cells transferred directly onto a slide, and examined in suspension, there is essentially no possibility that they are the artefact of handling. The appearance of CFs varied markedly between different cell lines: they were most prominent in RL cells, but were also seen in four other B-lymphoma cell lines, of a total of seven that were tested. Raji cells produced CFs, but were different from RL cells in that RL cells shed most of the bound Ab in CFs, while Raji cells retained a large fraction of the bound Ab in a JN spot (Michel and Mattes, 2002).

The results presented are descriptive, and much additional investigation is required in order to understand the biological and clinical significance of CF production. They appear to be relatively common in cell lines, but it is important to know if CFs are produced in primary tumours. Studies are in progress to determine if CFs are present in mouse xenografts of RL and Raji tumours after injection of anti-CD20 or anti-HLA-DR Abs. Since many patients receive therapy with anti-CD20 (rituximab), it may be possible to detect such structures in clinical specimens. Indeed, Kennedy *et al* (2004) recently described cell clusters in the blood of chronic lymphocytic leukaemia patients that had been infused (for 7 h) with rituximab. In these cell aggregates, which are strikingly similar to the clusters shown in Figure 5, B cells are clustered around ‘debris’ which contains a large amount of the injected Ab and complement component C3. Their data also suggested that the Ab and bound antigen were stripped from B cells, which therefore became CD20-deficient while continuing to circulate in the blood. This result is consistent with our data, since membrane staining decreased in intensity as bright CFs formed. While Kennedy *et al* (2004) did not observe similar events in *in vitro* experiments, after a 7 h incubation with Ab, and therefore argued that macrophage-lineage cells were necessary for CD20 stripping to occur, there are two possible explanations for this apparent inconsistency. First, primary CLL cells incubated *in vitro*, as used by Kennedy *et al* (2004), are likely to not behave like healthy cells *in vivo*, even though the incubation period is relatively short and cell death has not yet occurred. Second, there may be differences between CLL cells and the non-Hodgkin's lymphoma cell line RL, which was used in most of our experiments. Kennedy *et al* (2004) emphasised the role of complement in the process of CD20 removal from the cell surface, but did not provide direct evidence that complement was required. Our results were similar with IgG1 and IgG2a Abs, which strongly suggests that complement activation is not a significant factor, since IgG1 Abs do not activate complement (Goding, 1983). (In our experiments, the available complement would be from the foetal bovine serum in tissue culture medium; this would not be likely to have high complement activity, but may have some complement components.) Since the CFs contain certain Abs, but not all, there is a selective process for delivering Abs to this site, and it is important to test Abs to a wide range of other antigens. Both CD20 and MHC class II antigens are known to be enriched in membrane lipid rafts; for MHC class II antigens this is a spontaneous association (Anderson *et al*, 2000), while for CD20 this association is induced by Ab binding (Petrie and Deans, 2002). There is also evidence for a physical association of MHC class II antigens and CD20 (Léveillé *et al*, 1999).

These results explain previous paradoxical Ab uptake results, which were described in the first section. That is, in prolonged incubations of RL cells with saturating concentrations of radiolabelled 1F5, it was found that uptake of the Ab was much higher at 24 h than at 1 h, yet such an increase was not detected in immunofluorescence experiments by FACS analysis of individual cells. This result is expected if Abs are delivered primarily to CFs. The Abs present in the CFs pellet together with the cells, and therefore are detected as bound radioactive Abs. In contrast, such Ab uptake would not significantly affect the FACS results, since a small fraction of the cells have bound CFs, and many of the CFs are cell-free at the time of analysis. Such cell-free CFs may or may not be counted as cells, depending on their size, but even if they were counted they would be recorded as a small number of very bright objects, and hence would have little if any impact on the overall FACS profile.

It is uncertain whether the Ab is delivered to the CFs directly from the cell surface or after internalisation. Some Ab does go to intracellular vesicles, but it seems most likely that the CFs are produced by shedding of capped Ab. Although shedding is often cited as an alternative fate of capped Ab (Schreiner and Unanue, 1976; Loor, 1980) (in addition to the predominant endocytotic pathway), the shedding of large CFs as described herein has not been described previously, to our knowledge. Spontaneous shedding (in the absence of any ligand binding) of large CFs from viable cells has been described in certain circumstances (Black, 1980; Loor, 1980; Kaplan and Keogh, 1982). Such shedding has been attributed to the pinching off of microvilli or other membrane protrusions. Since the shedding in our studies was dependent on Ab binding, it is distinct from the types of spontaneous shedding that have been described. Fritzsching *et al* (2002) described the release of membranous microparticles, consisting of both exosomes and larger microvesicles, from T-cell lines upon activation. These microparticles selectively contained CD81, a tetraspanin; although CD20 is not in the tetraspanin family, it does share the property of having four transmembrane regions. We note also that circulating CD20 in the serum of non-Hodgkin's lymphoma patients was recently described (Giles *et al*, 2003); this antigen appeared to be in the form of small membrane fragments, and did not require Ab treatment for induction, but some relationship with the shedding process described here is possible.

Morphologically similar objects have been described previously, for many years, under the name of lymphoglandular bodies (LGBs) or Söderström bodies (Söderström, 1968; Flanders *et al*, 1993; Bangerter *et al*, 1997; Stern *et al*, 2001). These are well established in the pathology literature, although there is no previous report of their production by cultured cell lines, and also no report of Ab delivery to these structures. Lymphoglandular bodies are considered characteristic of B-cell lymphomas, and are an aid in identification of this tumour type. They are defined as large, cell-free cytoplasmic fragments, sometimes containing organelles, with a diameter ranging from 2 to 7 *μ*m in smears. Although the recent literature emphasises the presence of LGBs in tumours, there are older reports that they also occur in normal lymphoid tissue (Williamson, 1950; Söderström, 1968). While LGBs have been often dismissed as an artefact of handling (Söderström, 1968), this remains an open question. Although speculative, it seems possible, and perhaps likely, that the CFs described here are in fact LGBs.

A basic question is whether the CFs are naturally detached from the cells, or whether they are cytoplasmic extensions that become detached during handling procedures. If CFs form initially as filopodia-like extensions, which are intertwined with those of adjacent cells, then such extensions may be fragile, and may detach from the cells due to handling. Our results are not inconsistent with this possibility, since free CFs were seen only after washing the cells, and since intact, unwashed clusters had Ab localised only at the centre of the clusters. However, it seems unlikely that cytoplasmic extensions could be torn from a cell without killing that cell. Since the viability of our cell preparations was generally high, >95%, and was not affected by 1F5 binding, this possibility seems unlikely. In some experiments, dead cells were counterstained with propidium iodide: dead cells were never bound to CFs, and the cells bound to fluorescent CFs were essentially all viable cells. Electron microscopy may help to resolve this question. It should be emphasised, in any case, that the dispersal of cell clusters and CFs resulted from standard methods used to wash away unbound Ab. The development of CFs in the centre of cell clusters, in RL cell preparations, probably explains characteristics such as their heterogeneity in size, and the fact that a very small fraction of the cells have bound CFs. In the intact cell clusters staining appeared quite homogeneous, with essentially all of the cells being adjacent to a CF at the centre. The heterogeneity that appears after dispersal may thus reflect the fact that the CFs break apart unevenly, resulting in CFs of varying size, both cell-bound and free.
